# Active Inclusion Bodies in the Multienzymatic Synthesis of UDP-N-acetylglucosamine

**DOI:** 10.3390/ijms26199679

**Published:** 2025-10-04

**Authors:** Romana Köszagová, Klaudia Palenčárová, Jozef Nahálka

**Affiliations:** 1 Institute of Chemistry, Centre for Glycomics, Slovak Academy of Sciences, Dubravska Cesta 9, SK-84538 Bratislava, Slovakia; chemrore@savba.sk (R.K.); chemktal@savba.sk (K.P.); 2 Institute of Chemistry, Centre of Excellence for White-Green Biotechnology, Slovak Academy of Sciences, Trieda Andreja Hlinku 2, SK-94976 Nitra, Slovakia

**Keywords:** active inclusion bodies, multienzymatic synthesis, nucleotide triphosphate regeneration, UDP-N-acetylglucosamine one-pot synthesis

## Abstract

Bacterial inclusion bodies (IBs) are still generally considered to be waste products of recombinant protein production, despite various studies that have challenged this conventional view in the last two decades, and have been proposed for use as immobilized enzymes in vivo for biocatalysis. Current advances in genetic and molecular biology make it possible to perform multienzymatic reactions or enzymatic cascades to synthesize valuable products. When cascades need cofactor regener tion, it is difficult to use “cheap” whole cells or their lysates, and “expensive” enzyme purification is required. The capture of enzymatic activity into active IBs (aIBs), well-separable protein aggregates from cell lysate, could represent a usable compromise between purified enzymes and cell lysates. It is shown here that the combination of two polyphosphate kinases (PPKs) in the form of aIBs leads to almost 10-fold ATP regeneration and 100% UTP utilization without degradation into adenosine or uridine. PPKs have been combined with N-acetylhexosamine 1-kinase and N-acetylglucosamine-1-phosphate uridyltransferase to produce valuable UDP-N-acetylglucosamine, but the described approach could be used in various multienzymatic syntheses to avoid enzyme purification and ensure nucleotide triphosphate regeneration.

## 1. Introduction

In the past, bacterial inclusion bodies (IBs) were defined as inclusion structures revealed using electron microscopy. They were usually divided into two main groups based on the presence or absence of a surrounding membrane and were known as polyglucoside granules, polyphosphate granules, parasporal inclusions, poly-β-hydroxybutyrate granules, sulfur globules, and many other things [1]. The possibility of cloning DNA in vitro opened the door to the production of recombinant proteins, and the bacterium *Escherichia coli* became the main host organism for research, as it is most likely the cheapest organism to cultivate [2]. In general, approaches to expressing active soluble protein in *E. coli* result in a high amount of IBs composed predominantly of partially folded recombinant protein. Currently, bacterial inclusion bodies are more commonly defined as aggregates of recombinant proteins that occur as larger protein clusters that self-precipitate as insoluble materials [3]. Over the years, the notion of IBs as an undesirable byproduct has slowly but fundamentally changed to a desirable form for the expression of proteins/peptides toxic to host cells, e.g., antimicrobial peptides/proteins [4], or the expression of self-immobilized enzymes for biocatalysis [5]. In biotechnology, the general concept of active inclusion bodies (aIBs) is well established, especially in biocatalysis [6]. It has been shown that despite the much lower enzymatic activity of aIBs per mg of protein (specific activity) compared to that of the soluble enzyme, expression of the protein in *E. coli* usually achieves the same tolerable activity per g of cells [7]. Induction of aIBs is usually achieved by fusing the target protein with an appropriate aggregation tag. In our laboratory, we use the 20 kDa cellulose-binding domain (CBD) of the bacterium *Clostridium cellulovorans* [7] and LHS1 or dual LHS1 tags (10 or 20 amino acids) [8]. However, L6KD, GFII8, ELK16, 18AWT, TDoT, Aβ42, VP1, MalE31, and many others can be used for this purpose [6], and sometimes, their combination may be appropriate, e.g., the combination of L6KD and SUMO [9]. In summary, aIBs represent protein IBs generated during the overexpression of a recombinant protein, and the activity increases when the target protein is more folded and aggregation is achieved mainly through the fused aggregation tag.

UDP-*N*-acetyl-D-glucosamine (UDP-GlcNAc) is a nucleotide-monosaccharide substrate for some Leloir glycosyltransferases (Leloir GTs; Luis Federico Leloir received the Nobel Prize in 1970 for the discovery of nucleotide-sugar substrates). Compared to non-enzymatic coupling strategies that mostly rely on protecting group chemistry, Leloir GTs are known to bind a broad spectrum of unprotected sugar acceptors with excellent regio- and enantioselectivity. Despite the fact that Leloir GTs have been an important topic of interest in recent decades, no industrial use of Leloir GTs for the elongation or branching of glycoconjugates, oligosaccharides, and glycans has yet been scaled up to large volumes, unlike non-Leloir glycosyltransferases. The main limitation still remains the cost-effective production of NDP-sugar donors [10]. One example of Leloir GTs that utilize UDP-GlcNAc is hyaluronic acid synthase (HAS). HAS requires two substrates, UDP-GlcNAc and UDP-D-glucuronic acid, as hyaluronic acid (HA) is a linear heteropolysaccharide [11]. Both substrates are expensive, so microbial fermentation is currently the dominant method of HA production, despite providing limited options for controlling the molecular weight (MW) of the final product. HA is active in various physiological and pathological processes, and molecular weight (MW) is a key determinant of this activity. Enzymatic synthesis of HA in vitro could represent a good option for controlled and monodisperse synthesis of HA under defined conditions; however, as mentioned above, it requires inexpensive UDP-GlcNAc [11]. Inexpensive UDP-GlcNAc could also support research into O-linked N-acetylglucosamine modification of protein (O-GlcNAcylation) and drug development in this light. O-GlcNAcylation facilitates vascular repair, and conversely, the suppression of O-GlcNAcylation increases cellular vulnerability to oxidative stress, which is a major factor in aging [12,13].

Functional and structural characterization of N-acetylglucosamine-1-phosphate uridyltransferase (GlmU) from *Staphylococcus aureus* revealed redox-sensitive acetyltransferase activity, with GlmU being the enzyme responsible for UDP-GlcNAc synthesis. GlmU is a bifunctional enzyme, catalyzing the Mg^2+^-dependent reaction of UTP with N-acetylglucosamine 1-phosphate (GlcNAc-1-P) to form UDP-GlcNAc and pyrophosphate (PPi) and providing acetyltransferase activity on α-D-glucosamine 1-phosphate (GlcN-1-P) to form GlcNAc-1-P [14]. Fifteen years ago, an elaborate pathway for the preparative synthesis of UDP-GlcNAc was achieved using a two-step enzymatic reaction that combined GlmU and N-acetylhexosamine 1-kinase (NahK) from *Bifidobacterium longum* [15]. NahK catalyzes the ATP/Mg^2+^-dependent phosphorylation of N-acetylglucosamine (GlcNAc), which provides GlcNAc 1-P for GlmU. Fusion of both enzymes [16] and optimized reaction conditions [17] slightly improved the reaction. Further improvement was achieved by the introduction of the polyphosphate/PPK3 system, a nucleotide triphosphate generation system for UTP and ATP [18]. PPK3 is a type 2 subclass I polyphosphate kinase from *Ruegeria pomeroyi*, the first PPK that prefers pyrimidine over purine nucleotide diphosphate but is also capable of accepting both, so it can be used efficiently to generate UTP and ATP [19,20]. NahK accepts N-acetylgalactosamine and GlmU N-acetylgalactosamine-1-phosphate, so the same system was recently applied to the more expensive UDP-N-acetylgalactosamine and, given that uridine was used as the starting substrate instead of UTP and considering the overall yields, the authors achieved the best current results [21]. Another important fact is that GlmU releases inhibitory PPi during the reaction, so an additional enzyme (inorganic pyrophosphatase, PPA) is required in the system to degrade PPi [15,16,17,18,21]. Importantly, substitution of PPA with PPK2 from *Staphylococcus epidermidis* (SePPK), which is capable of generating ATP from ADP and PPi, has recently been proposed to exploit the high-energy phosphoanhydride bond of PPi and thereby enhance UDP-GlcNAc biosynthesis [22].

In summary, active inclusion bodies represent insoluble protein aggregates of recombinant protein that retain enzymatic activity and can be considered immobilized enzymes in vivo. aIBs offer the advantage of cost savings over purified and subsequently immobilized enzymes, being directly separated from the host cell lysate as insoluble residues. aIBs also offer an advantage in multienzyme reactions over whole cells or cell lysates because they separate recombinant enzymes well from host cell enzymes that may negatively affect the designed cascade reactions. The presented study shows that aIBs of recombinant polyphosphate kinases are well separated from cellular phosphatases that accumulate in host cells during recombinant overproduction of the kinases.

**Figure 1:**
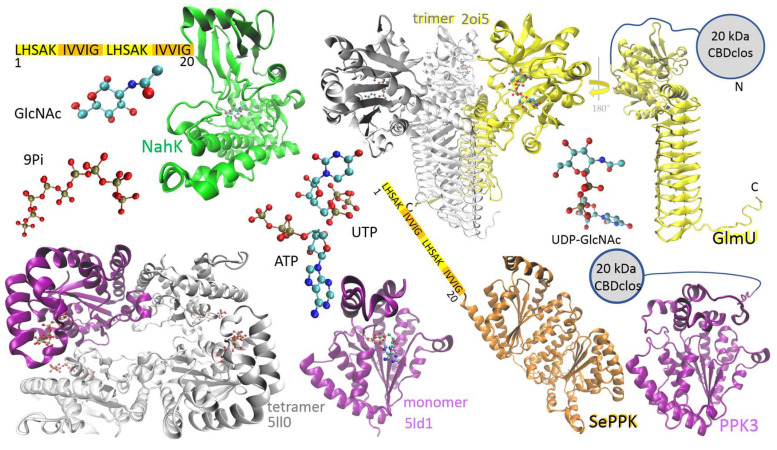
Enzymes used for synthesis of UDP-GlcNAc: N-acetylhexosamine 1-kinase (NahK) from *Bifidobacterium longum* (green structure, PDB ID code 4ocj), N-acetylglucosamine-1-phosphate uridyltransferase (GlmU) from *Escherichia coli* (trimeric structure, PDB ID code 2oi5) and *Bifidobacterium longum* (yellow structure, AlphaFold-predicted), PPK2-I from *Francisella tularensis* (tetrameric structure, PDB ID code 5ll0), PPK2-I from *Meiothermus ruber* (purple monomer, PDB ID code 5ld1), SePPK (orange structure, AlphaFold-predicted), and PPK3 (purple structure, AlphaFold-predicted). NahK and SePPK are N-terminally fused with a dual LHS1 tag (20 amino acids) designed for pull-down into aIBs. GlmU and PPK3 are N-terminally fused with the 20 kDa CBD of the bacterium *Clostridium cellulovorans* (CBDclos), which serves as an aggregation module to make aIBs.

UDP-N-acetyl-D-glucosamine is a valuable substrate for some Leloir glycosyltransferases, and there is still interest in reducing its price. In recent decades, intensive research into the enzymatic synthesis of UDP-GlcNAc has led to a system in which polyphosphate generates ATP and UTP and UDP-GlcNAc is synthesized by a combination of NahK and GlmU. In this study, we demonstrate the successful production of UDP-GlcNAc using a multienzymatic cascade composed of GlmU [23], NahK [24], PPK3 [20], and SePPK [22] (Figure 1), all expressed as aIBs, thus offering a cost-effective and efficient method for nucleotide triphosphate regeneration and product synthesis.

## 2. Results

**Figure 2:**
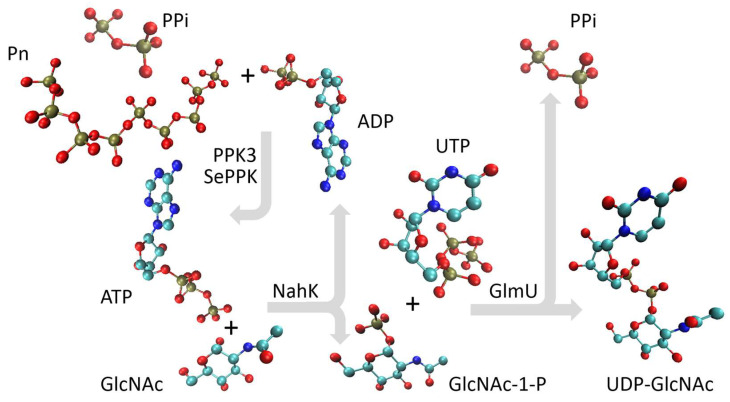
A schematic representation of the multienzyme cascade for the synthesis of UDP-GlcNAc. PPK3 and SePPK regenerate ATP through transfer of the phosphate group from polyphosphate (Pn) and pyrophosphate (PPi) onto the phosphate group of ADP; then, the phosphate group is transferred from ATP onto C1 of GlcNAc by NahK to form GlcNAc-1-P, and subsequently, GlmU condenses GlcNAc-1-P with UTP to form PPi and UDP-GlcNAc.

NahK from *Bifidobacterium longum*, which catalyzes the phosphorylation of GlcNAc to GlcNAc 1-P, is a monomer in solution, and its polypeptide folds into a crescent-like architecture subdivided into two domains by a deep cleft, and the sugar-binding site is typically oriented into the cleft (Figure 1, green structure) [24]. The enzyme was N-terminally fused with the dual LHS1 tag (20 amino acids) designed for pull-down into aIBs [8]. The bifunctional enzyme GlmU, performing the acetyltransferase activity on GlcN-1-P to form GlcNAc-1-P and subsequent GlcNAc-1-P uridyltransferase activity to form UDP-GlcNAc, makes a trimeric structure, the acetyltransferase active site is contained within the C-terminal β-helical domain, and the uridyltransferase active site is contained within the N-terminal globular uridyltransferase domain (Figure 1, yellow structure) [23]. The GlmU monomer was N-terminally fused with the 20 kDa CBD of the bacterium *Clostridium cellulovorans* (CBDclos), which served as an aggregation module to make aIBs [7]. PPK2 enzymes are divided into three subclasses: PPK2-I (which phosphorylates NDP); PPK2-II (which phosphorylates nucleotide monophosphate (NMP)); and PPK2-III (which phosphorylates both NMP and NDP). The class PPK2-I consists of one-domain enzymes, while the PPK2-II and -III classes includes both one- and two-domain proteins [25]. PPK3 is a one-domain PPK2-I from *Ruegeria pomeroyi* (Figure 1, purple structure), and SePPK is a two-domain PPK2-III from *Staphylococcus epidermidis* (Figure 1, orange structure). Because of its high degree of similarity to PPK2-I from *Meiothermus ruber* and *Francisella tularensis*, PPK3 probably forms a tetrameric structure (Figure 1). In this study, PPK3 was N-terminally fused with the 20 kDa CBDclos and SePPK with the dual LHS1 tag (20 amino acids). As already mentioned in the introduction (1), PPK3 and SePPK were selected over other variants because PPK3 prefers pyrimidine over purine nucleotide diphosphate and SePPK is able to consume inhibitory PPi. In the four-enzymatic reaction, the phosphate group is transferred from ATP onto C1 of GlcNAc by NahK to form GlcNAc-1-P, and subsequently, GlmU condenses GlcNAc-1-P with UTP to form pyrophosphate and UDP-GlcNAc. PPK3 and SePPK regenerate ATP by transferring the phosphate group from polyphosphate (Pn) and pyrophosphate onto the phosphate group of ADP (Figure 2).

**Figure 3:**
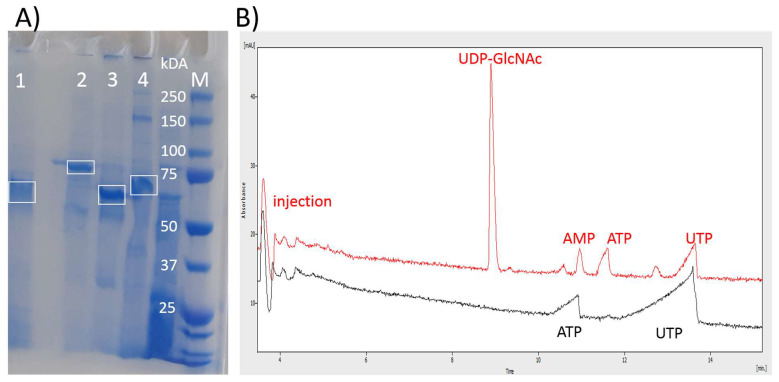
Presentation of the purity and activity of the aIBs used for synthesis of UDP-GlcNAc. (**A**) SDS-PAGE shows that aIBs are composed predominantly of the recombinant enzyme: 1—NahK; 2—GlmU; 3—PPK3; 4—SePPK. (**B**) A CE electropherogram demonstrating the activity of the aIBs. Black line—the start of the reaction with 2 mM ATP and 10 mM UTP; red line—UTP is converted into UDP-GlcNAc, 15 h of reaction. There is no residual ATP or UTP degradation activity (adenosine and uridine have peaks between the injection signal and UDP-GlcNAc).

**Figure 4:**
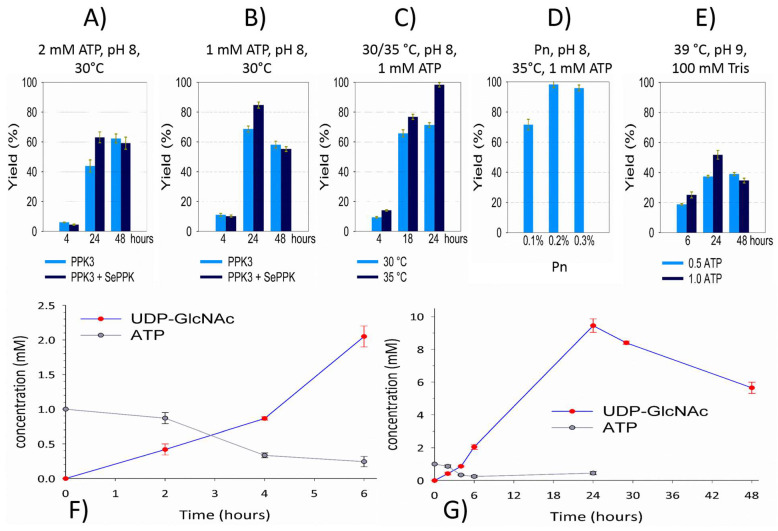
Optimization of the reaction system for UDP-GlcNAc. aIBs from 5 mg of lyophilized cells were resuspended in 250 μL of buffer. NahK (4 μL), GlmU (4 μL), PPK3 (5 μL), and SePPK (5 μL) were resuspended in a fresh reaction mixture: 50 mM GlcNAc, 10 mM UTP, 1 mM ATP, 0.2% Pn, 20 mM MgCl_2_, and 50 mM Tris, pH = 8. Reactions were carried out at 35 °C with shaking at 300 rpm (using a ThermoMixer C Eppendorf). Different conditions are shown in the figure (**A**–**E**). Optimization of the reaction system was performed in a volume of 100 μL (**A**–**E**) and finally in a volume of 500 μL (**F**,**G**).

After the lysis of recombinant *E. coli* cells, the aIBs were washed with the buffer and directly used for SDS-PAGE (Figure 3A) and the activity test (Figure 3B). Compared to immobilized metal affinity chromatography (IMAC)-purified soluble enzymes [21], aIBs show more impurities, but compared to the cell lysates often directly used for enzymatic transformations, aIBs represent a good compromise between “cheap” lysates and “expensive” purified enzymes. Importantly, differently from cell lysates, the aIB mixture of these four enzymes did not show residual ATP or UTP degradation activity (adenosine and uridine have peaks between the injection signal and UDP-GlcNAc). As shown in the figure, no adenosine or uridine was observed, and UDP-GlcNAc was synthetized in the reaction mixture of the initial test (Figure 3B). In the test and subsequent experiments, the reaction mixture was in 50 mM Tris and consisted of 0.2% Pn, 20 mM MgCl_2_, 2 mM ATP, 50 mM GlcNAc, and 10 mM UTP. The pH was adjusted using KOH to 8, and the reaction was performed in a minimal volume (100 µL) with an excess of aIBs (Figure 4A). As was mentioned in the introduction, GlmU releases inhibitory PPi during the reaction, so previously, an additional enzyme (inorganic pyrophosphatase, PPA) has been required in the system to degrade PPi [15,16,17,18,21]. Li and co-authors recently introduced SePPK to utilize the high-energy phosphoanhydride bond of PPi and thereby enhance UDP-GlcNAc biosynthesis [22]. When testing the effect of SePPK, a decrease in UDP-GlcNAc was indeed observed when SePPK was not used (Figure 4A,B). ATP is not as expensive a substrate as UTP, and although NahK prefers ATP over UTP, it is able to use UTP to phosphorylate GlcNAc, so we started with a ratio of 2 mM ATP to 10 mM UTP. Surprisingly, however, reducing ATP to 1 mM led to a much better result (Figure 4A,B). aIBs represent incompletely folded proteins and are usually more sensitive to higher temperatures compared to soluble forms; therefore, we initially opted to perform the reaction at 30 °C, but increasing the temperature to 35 °C resulted in the complete conversion of UTP into UDP-GlcNAc (Figure 4C). In this study, a cheap polyphosphate (Pn) of an unknown molecular weight was used, and the ratio between Pn and Mg^2+^ was set to 0.2% and 20 mM based on our laboratory experience. Reaction testing confirmed this setting (Figure 4D). Despite the production of UDP-GalNAc, optimization of the reaction conditions for purified enzymes led to a higher temperature, a higher pH, and stronger buffer and even lower ATP, but testing the aIBs under these conditions resulted in half yields (Figure 4E). Based on the experiments performed, the optimal reaction conditions for the aIB enzymes were set to 50 mM Tris, 0.2% Pn, 20 mM MgCl_2_, 1 mM ATP, 50 mM GlcNAc, and 10 mM UTP at pH 8 and a temperature of 35 °C. Finally, we tested how much the reaction volume could be increased at the same concentration of aIBs, which, as mentioned, were in excess (4 µL of NahK, 4 µL of GlmU, 5 µL of PPK3, and 5 µL of SePPK). As shown, it was possible to increase the reaction volume fivefold (500 µL) and maintain the aIB enzymes at the same concentrations (Figure 4F,G).

## 3. Discussion

Industrial biocatalysis can be performed using whole cells, permeabilized cells, cell lysates, and purified enzymes. Whole cells can be genetically engineered to produce value-added chemicals, but this approach must deal with metabolic byproducts and reduced control over metabolic enzyme networks, so whole-cell catalysts or permeabilized cells are mainly used for simpler biotransformations. Cell-free in vitro systems offer much better possibilities for designing and controlling multienzyme cascades. Advances in molecular biology and genetic engineering have enabled the facile production of recombinant cell-free extracts. These inexpensive preparations can be used to catalyze multistep enzymatic reactions without the limitations of cellular toxicity or the cost and complexity associated with the production of purified enzymes [26]. However, recombinant cell-free extracts have a more difficult time recovering bioenergy in the form of nucleotide triphosphates (NTPs) or other cofactors compared to enzymatic cascades from purified enzymes because enzymes of entire metabolic pathways are present in cell lysates and the consumption of NTPs is faster than their regeneration. The in vivo aggregation of recombinant enzymes allows for their easy separation from the cell lysate, and although their purity does not reach that of IMAC-purified enzymes [21], this study shows that they can be used to regenerate ATP/UTP without the consumption of ATP/UTP and degradation into adenosine/uridine by contaminating enzymes from host cells (Figure 3).

In recent years, the polyphosphate–polyphosphate kinase (Pn/PPK) system has emerged as a promising, cost-effective, and scalable technique. It utilizes Pn as a phosphate donor and PPK to phosphorylate nucleotide diphosphate (NDP) into NTP (Figure 2). Pn is an ancient prebiotic polymer found from bacteria to humans and plays a significant role in many biological processes—from storing bioenergy and phosphates and regulatory processes to influencing cellular metabolism, growth, and differentiation. No other molecule concentrates so much biochemically usable energy [27]. Although *E. coli* has only one PPK1 gene, some bacteria encode multiple PPK genes: for example, *Ruegeria pomeroyi* encodes three genes, which were biochemically characterized in our laboratory ten years ago [20]. Currently, PPKs are divided into PPK1, PPK2-I, PPK2-II, and PPK2-III. These are single- and double-domain PPKs, with the single-domain PPK2-I from *Ruegeria pomeroyi* (PPK3) and the double-domain PPK2-III from *Staphylococcus epidermidis* (SePPK) used as aIBs in this study (Figure 1). PPK3 is ideal for generating UDP-monosaccharide substrates for Leloir GTs because most Leloir GTs prefer UDP-sugar and PPK3 prefers UDP [20]. PPK3 has already been used for multienzymatic synthesis of UDP-GlcNAc and UDP-GalNAc with IMAC-purified enzymes [18,21]. However, in these studies, inorganic pyrophosphatase (PPA) had to be used to hydrolyze the inhibitory PPi generated by GlmU [18,21]. SePPK has already been used for the synthesis of UDP-GlcNAc by IMAC-purified enzymes [22]. SePPK is ideal for ATP regeneration because it is a PPK2-III enzyme [28] that catalyzes the phosphorylation of both AMP and ADP and therefore “slows down” the degradation of ATP into adenosine by accompanying enzymes from the host cell. It has high enzymatic activity and preferentially utilizes AMP [29]. Furthermore, SePPK has been shown to be among the PPKs that are able to utilize inhibitory PPi to regenerate ATP [29,30]. Similar to some bacteria, we propose combining PPKs in this study. When a combination of PPK3 and SePPK is used for UDP-GlcNAc synthesis, no degradation of ATP/UTP into adenosine/uridine is observed (Figure 3), and 90–100% of UTP is condensed into UDP-GlcNAc within 24 h (Figure 4C,D,G).

As mentioned in the Introduction (Section 1), enzymatic synthesis of UDP-GlcNAc has been extensively investigated in the past two decades, with several synthetic routes for UDP-GlcNAc established based on its biosynthetic pathways [31,32], but an improved route for the preparative synthesis of UDP-GlcNAc has been achieved through two-step enzymatic reactions using NahK and GlmU [15] or a one-step reaction with the fusion enzyme NahK-GlmU [16]. Synthesis was improved further by introducing the Pn/PPK3 system and using uridine as the starting substrate [18,21]. Starting from inexpensive uridine prevented UTP degradation, and the system could potentially be used for whole-cell reactions. However, our study shows that an enzymatic module composed of only four enzymes, which can easily be separated from the cell lysate in the form of aIBs, achieves almost 100% conversion of UTP into UDP-GlcNAc (Figure 4C,D,G). This means that the aIBs of recombinant PPK3 and SePPK are well separated from the cellular phosphatases that host cells accumulate during recombinant production of the aforementioned kinases. ATP is cheap compared to other NTPs, but its concentration above 1 mM is inhibitory for some reason (Figure 4A,B), so it must be regenerated (10-fold). In summary, it can be concluded that the Pn/PPK3/SePPK module in the aIB form is functional and could be used for ATP/NTP regeneration and the cost-effective production of UDP-GlcNAc and other NDP-sugar donors.

Not only are NTP cofactors required for nucleotide-activated sugars in the biochemistry of Leloir glycosyltransferases [10,11] but ATP/NTP cofactors are involved in a vast array of diverse biological processes, such as the synthesis of nucleic acids, lipids, and proteins. Cell-free protein synthesis [33,34] or enzymatic activation of alcohols and carboxylic acids [35] may be examples of potential scientific and industrial implementations of the Pn/PPK3/SePPK module.

aIBs can be applied in various process designs, and several approaches can be used for further improvements. Optimization of multienzymatic syntheses can include the yield (g product/g substrate), conversion (mol product/mol substrate), reaction rate (mol/s), or space–time yield (STY, g/L⋅h), using various in silico and experimental methods [36]. For example, in the case of UDP-GlcNAc production, a comparison of batch and flow systems showed that the STY is 0.212 for soluble enzymes and 0.096 for immobilized enzymes in the batch process, but in the flow process with immobilized enzymes, the STY increases with flow rate from 1.9 to 2.37 [37]. aIBs represent in vivo immobilized enzymes and achieved an STY of 0.253 in a small-volume batch in this study. Nevertheless, it was shown recently that a repetitive batch mode design can lead to an exceptionally high STY of 9.9 g/L⋅h [38]. In our opinion, there are two options: aIBs can be used once as a cheaper alternative to purified soluble enzymes in a batch process, as shown here, or they can be magnetized and used in a repeated batch process [39] or in a flow process. Enzymes aggregated with LHS1 appear to be more easily solubilized from aIBs [40] but have much higher activity compared to that of enzymes aggregated with CBDclos (see Section 4.1), which are less active but are more compact and can be magnetized for continuous or repeated applications [39].

## 4. Materials and Methods

### 4.1. Cloning, Expression, and Isolation of aIBs

Codon optimization for *E. coli*, gene synthesis, and cloning (NdeI/BamHI) with the pET-30b (+) plasmid were performed using GenScript. The 20-amino-acid LHSAKIVVIGLHSAKIVVIG tag [8] was inserted behind the initial methionine into the amino acid sequence of NahK (https://www.rcsb.org/fasta/entry/4OCK/display; accessed on 3 November 2025) and SePPK (https://www.kegg.jp/entry/sep:SE_2128; accessed on 3 November 2025). In the case of PPK3 and GlmU, genes were propagated using PCR and inserted into the pET-34b (+) plasmid with am N-terminal CBDclos fusion sequence. A terminating stop codon was added to the insert of all enzymes, and therefore, no His-tag was included. Chemically competent *E. coli* BL21 (DE3)T1R cells were transformed with the plasmid and cultivated on kanamycin/LB/agar plates. The colonies were regrown in 30 mL LB medium supplemented with kanamycin (30 μg/mL) for approximately 20 h at 32 °C; then, 15 mL of the solution was transferred into a 300 mL flask with 100 mL of LB media and incubated for 4 h at 37 °C with shaking at 150 rpm. Recombinant expression was induced by adding an inductor (isopropyl β-d-1-thiogalactopyranoside; 400 μM) for 20 h at 15–20 °C, with shaking at 150 rpm. After centrifugation (45 min, 9700× *g*, 4 °C, using an Avanti JXN-30 centrifuge, Beckman Coulter, Brea, CA, USA), the biomass was suspended in a minimum volume of water, frozen, and lyophilized. The lyophilized cells were stored at −25 °C until further use. Lyophilized cells were lysed twice in BCL (CelLytic B Cell Lysis Reagent; Sigma-Aldrich, St. Louis, MO, United States) at a ratio of 1 mg of cells/100 μL of BCL solution. After centrifugation of the lysate (5 min, 16,060× *g*, 4 °C, using a Hettich centrifuge mikro 220r, Kirchlengen, Germany), cell debris was washed three times through centrifugation using the same ratio of Tris-HCl buffer (50 mM, pH = 7.5). After the final centrifugation step, the IBs were resuspended in half the volume of Tris-HCl buffer (aIBs from 5 mg of lyophilized cells in 250 μL of buffer) and stored at 4 °C. The purity and quality of the aIBs were measured through SDS-PAGE. The initial enzyme activities of the aIBs were as follows: NahK—604.2 U/g DCW (Dry Cell Weight); GlmU—20.2 U/g DCW; PPK3—13.4 U/g DCW; SePPK—409.9 U/g DCW; NahK—0.01208 U/μL isolate; GlmU—0.000404 U/μL isolate; PPK3—0.000268 U/μL isolate; SePPK—0.00820 U/μL isolate. The enzyme unit (U) was defined as the amount of the enzyme that catalyzed the conversion of one micromole of substrate per minute. For NahK, 1 mM ATP and 50 mM GlcNAc were the substrates, 1 mM UTP and 1 mM GlcNAc1P were the substrates for GlmU, 1 mM ADP and 0.2% Pn were the substrates for PPK3, and 1 mM AMP and 0.2% Pn were the substrates for SePPK. Reactions were carried out at 35 °C in 50 mM Tris with 20 mM MgCl_2_, at a pH = 8. SePPK did not show activity with the 1 mM ADP and 0.2% Pn substrates under the same conditions as those for the 1 mM AMP and 0.2% Pn substrates.

### 4.2. Multienzymatic Synthesis of UDP-GlcNAc by the aIBs

NahK (4 μL), GlmU (4 μL), PPK3 (5 μL), and SePPK (5 μL) were resuspended in a fresh reaction mixture: 50 mM GlcNAc, 10 mM UTP, 1 mM ATP, 0.2% Pn, 20 mM MgCl_2_, and 50 mM Tris, pH = 8. Reactions were carried out at 35 °C with shaking at 300 rpm (ThermoMixer C Eppendorf, Ryde, Australia). Optimization of the reaction system for UDP-GlcNAc was performed in a volume of 100 μL (Figure 4A–E) and finally in a volume of 500 μL (Figure 4F,G). Initial experiments were performed with 2 mM ATP at 30 °C (Figure 4A,B). Reactions were monitored through capillary electrophoresis (CE).

### 4.3. Capillary Electrophoresis

The samples were diluted in buffer (100 mM Tris, 30 mM MgCl_2_, pH = 8), cleared/degassed through centrifugation (14,000× *g*, 5 min, using a Hettich centrifuge mikro 220r) and analyzed using CE (Figure 3). CE was performed on a PrinCE Next/800 system equipped with a fused silica capillary 70/30/40. The running buffer was 25 mM sodium tetraborate, pH = 9.4. The detector was set to 254 nm.

## Data Availability

Data are contained within the article.
